# Supplementary material for: How human runners regulate footsteps on uneven terrain
Source: eLife. 2023 Feb 22;12:e67177. doi: 10.7554/eLife.67177 (PMC10030110; doi:10.7554/eLife.67177)
Supplement: Figure 3—source data 1. [file elife-67177-fig3-data1.pdf]

Figure 3—source data 1. Footstep counts for each subject on all terrain.

subject	flat	uneven I	uneven II
1	363	321	448
2	228	78	473
3	473	131	436
4	373	471	519
5	366	557	109
6	224	160	327
7	218	398	442
8	503	489	479
9	477	390	392
